# *Xenobennettella coralliensis* a new monoraphid diatom genus characterized by the alveolate sternum valve with cavum, observed from coral reef habitats

**DOI:** 10.7717/peerj.13977

**Published:** 2022-10-19

**Authors:** Andrzej Witkowski, Catherine Riaux-Gobin, Adrian Kryk, Tomasz Płociński, Izabela Zgłobicka, Krzysztof Kurzydłowski

**Affiliations:** 1 Institute of Marine and Environmental Sciences, University of Szczecin, Szczecin, Poland; 2 Laboratoire d’Excellence ‘CORAIL’, University of Perpignan, Perpignan, France; 3 CNRS-UPVD-EPHE, USR3278 CRIOBE, PSL Research University, Perpignan, France; 4 Faculty of Material Science and Engineering, Warsaw University of Technology, Warsaw, Poland; 5 Faculty of Mechanical Engineering, Institute of Mechanical Engineering, Białystok University of Technology, Białystok, Poland

**Keywords:** *Xenobennettella*, New diatom, Cavum, Nukutavake, Juan de Nova, Coral reef, Heterovalvy, Achnanthidiaceae, Tuamotu Archipelago, Mozambique channel

## Abstract

During a survey of benthic diatoms from the coral reefs of the Indian Ocean (Scattered Islands) and Pacific Ocean (Tuamotu Archipelago), an interesting monoraphid diatom was observed and examined by light microscopy and various electron microscopy methods including Focus Ion Beam milling. Our thorough analysis revealed the similarity of this diatom to *Bennettella* R.W.Holmes, which we reference in the name: *Xenobennettella* Witkowski & Riaux-Gobin gen. nov., with *Xenobennettella coralliensis* Witkowski & Riaux-Gobin sp. nov. as the generitype. The type habitat for this new species is the sublittoral coral reef of Juan de Nova in the Mozambique Channel. The sternum valve of the new genus is characterized by an alveolate ultrastructure with the rim of the alveola opening along the valve margin, resembling the sternum valve of *Bennettella*. Internally, *Xenobennettella* differs from the latter by possessing a cavum (horseshoe-shaped chamber) on one side of the valve, in a central axial position. The raphe valve of *Xenobennettella* has small, marginal, apically elongate chambers, which are internally delineated by transapical ribs that are very similar to *Bennettella*. However, the raphe in the new genus is different from the latter, resembling some *Cocconeis* and *Planothidium* with internal raphe endings bent in the opposite direction, while resembling some *Planothidium* taxa externally by ending on the apical part of the mantle. This contrasts to *Bennettella*, which has a unique raphe system, with external raphe endings below the apices, a prominent axial structure and a transapically expanded central area. Likewise, the external surface of *Bennettella* is different from that of the new genus with a complex mantle structure and biseriate striae. In *Xenobennettella*, the valve mantle of the raphe valve is simple and perforated by areola. The transapical striae occur in the valve margin and the axial area is ornamented along its course with a single row of densely packed areola on both sides. The characteristics of the raphe valve and alveolate sternum valve place the new genus among the Achnanthidiaceae.

## Introduction

Biodiversity at various kinds of levels is essential for upkeeping the life in our planet. Diversity of all organisms provide healthy ecosystems for humans but the more we research biodiversity the more we notice how still limited is our knowledge. The most important to any biodiversity study is the identification of species. Therefore, describing new organisms, their classification and revision of the existing ones is crucial for a better understanding of the earth’s ecosystem biodiversity. In this article, we present and describe a new monoraphid diatom genus with cavum on alveolate sternum valve (SV) and monolayered raphe valve (RV) with the first species *Xenobennettella coralliensis* Witkowski & Riaux-Gobin sp. nov. Diatoms are unicellular, photoautotrophic eukaryotes characterized by their siliceous (opaline) exoskeleton. With the number of genera exceeding 1200 (Fourtanier & Kociolek, 2009), and species estimated as ca. 100,000 (Mann & Vanormelingen, 2013), diatoms are widespread in freshwater, terrestrial and marine habitats and as primary producers play an important role in the silica, carbon and oxygen global geochemical cycles (Field et al., 1998). In shallow water aquatic habitats, diatoms play an important role in the ecosystem services at the base of the trophic chain (Cox et al., 2020). The above aspects make diatoms important for taxonomists, ecologists and geochemists.

Whereas the RV of new species was characterized by LM and SEM, to reconstruct the 3D structure of the SV, we have milled it at the nanoscale with Focus Ion Beam (FIB). This novel method of microscopy became in recent years important for not only material engineering or imaging specialists but also for diatomists (*e.g.*,  Ehrlich et al., 2016; Zgłobicka et al., 2017; Zgłobicka et al., 2021). Witkowski et al. (2020) showed that FIB is a powerful tool and can be used in the taxonomic assessment of various diatom genera.

Use of nanosectioning methods is specially recommended in the 3-D structures with variable degrees of mineralization where solid and chambered silica ultrastructures occur. In such diatoms with chamber (cavum), the use of nanoscale cuts through the valve can provide easy and detailed assessment of the 3-D variation in the spatial structure.

Numerous diatom taxa possess valves composed of alveolae *i.e.*, the transapical striae are formed as part of an elongate chamber (Round, Crawford & Mann, 1990). Externally, the alveolae are covered by a perforated plate while internally closed with a plain siliceous plate. In addition, the internal wall has at least one elongate opening (foramina) positioned either in the valve center or along its margin. The best examples of alveolae in pennate diatoms are illustrated in taxa belonging to *Caloneis* and *Pinnularia* (Krammer & Lange-Bertalot, 1986; Round, Crawford & Mann, 1990). Among monoraphid diatoms there are numerous species characterized with alveolate valve structures. Although *Cocconeis scutellum* (the generitype of *Cocconeis* Ehrenberg) has monolayered valves, the genus includes the section Alveolatae (De Stefano & Romero, 2005; De Stefano, Romero & Totti, 2008), comprised of taxa with a monolayered RV and alveolate SV. The alveolate valve structure is also found in some strictly epizoic monoraphid taxa *e.g.*, *Bennettella* R.W.Holmes and *Epipellis* R.W.Holmes. There is one significant difference between the valve structure of the two epizoic genera: whereas *Bennettella* has an alveolate SV, *Epipellis* has both SV and RV alveolate (Denys & De Smet, 2010; Ferrario et al., 2019; Holmes, 1985).

The newly described *Xenobennettella* most important taxonomic characteristics are the presence of the cavum on the SV and the apical raphe ends bent in the same direction on the RV. Among monoraphid genera, there is a series of species characterized with a cavum on the SV including some *Planothidium* and all *Gliwiczia* species. Whereas *Planothidium* taxa possess a cavum only on the SV (Krammer & Lange-Bertalot, 1991), the second genus possess this remarkable structure on both valves (Kulikovskiy, Lange-Bertalot & Witkowski, 2013). To the best of our knowledge, *Xenobennettella* is the first described marine monoraphid diatom genus characterized with a cavum (cf. Krammer & Lange-Bertalot, 1991; Kulikovskiy, Lange-Bertalot & Witkowski, 2013). Likewise, this is the first cavum-bearing diatom genus with alveolate SV. Use of FIB allowed us to reveal the alveolate structure of the *Xenobennettella* SV valve structure including the alveola opening (named foramina) on the margin on one side and the solid and plain silica plate within the sternum and the valve part shaded with the cavum.

## Materials & Methods

### Fieldwork

**Figure 1:**
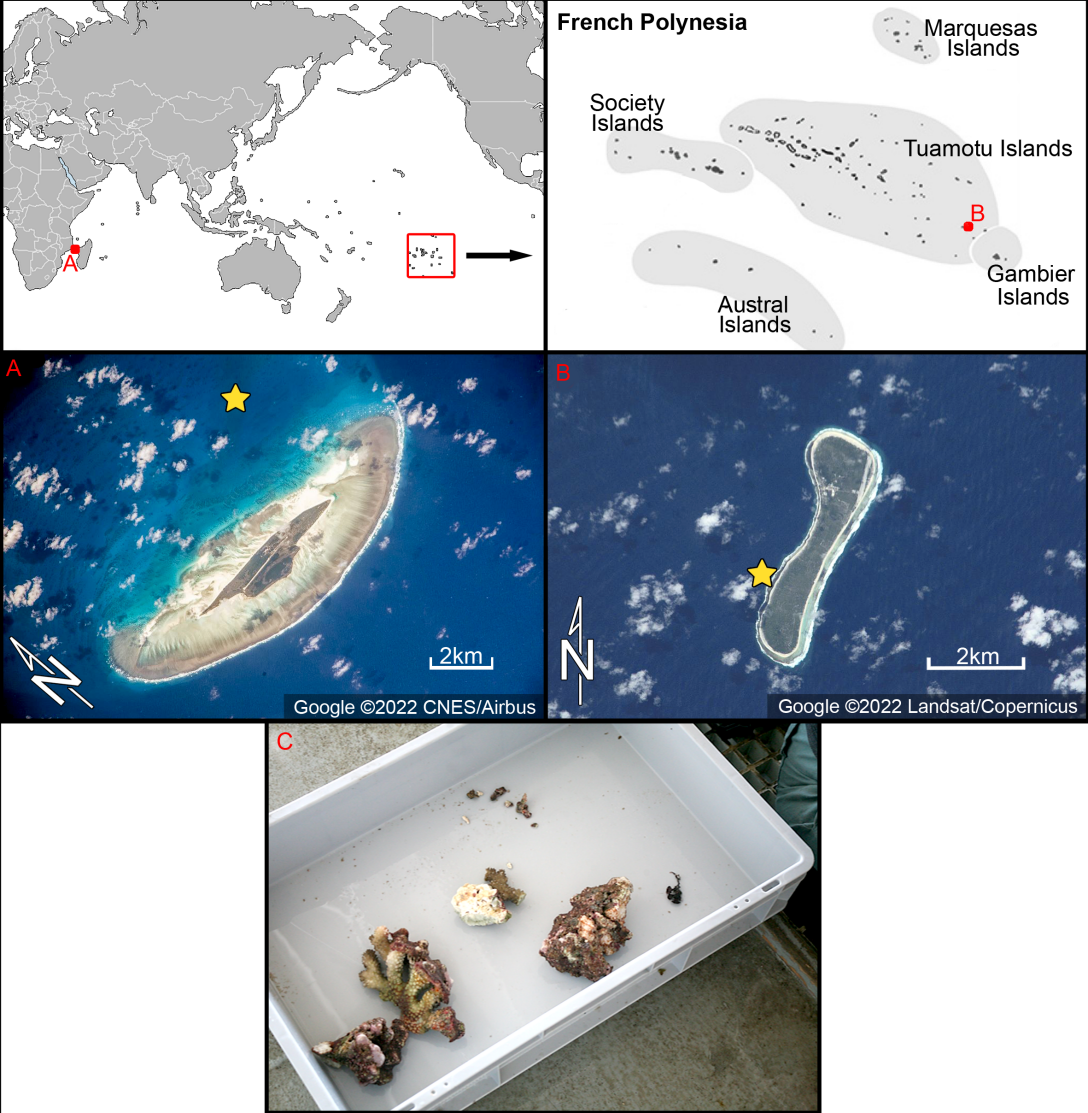
Location of the study areas with satellite images. (A) Juan de Nova coral island, Western Indian Ocean. (B) Nukutavake atoll at Tuamotu archipelago, South Pacific. Yellow stars indicate location of samples used in this study. Figure source credit: Google. (C) Photograph of sampled microhabitat from Juan de Nova–corals taken by scuba diver. Photo source credit: Andrzej Witkowskiv.

In the Western Indian Ocean, fieldwork was carried out on the 26th–29th of April 2009 in Juan de Nova coral island (Fig. 1). Coral samples were retrieved by scuba diving and preserved in alcohol (GPS data 16°59.34′S, 42°47.37′E) collected on April 28th 2009 from a water depth of 20 m.

In the South Pacific, samples were collected from Nukutavake, a small atoll located in the eastern part of the Tuamotu archipelago (Fig. 1). Nukutavake (GPS data 19°16.83′S 138°47.11′W) is 5 km in length, 13.5 km^2^ in surface area with a sediment-filled lagoon. Samples were obtained by a scuba diver on the 22nd October to 3th November 2016, on the reef slope (<5 m deep) of the atoll. Small amounts of detritus coral sands or epiphytes on dead corals were collected in small vials (50 ml) and preserved with alcohol.

### Light microscopy (LM)

For light microscopy, the samples were washed with distilled water to remove salts, treated with 30% H_2_O_2_ (hydrogen peroxide) for 2 h at 70 °C to remove organic matter, followed by the addition of ca. 10ml of 10% HCl to remove calcium carbonate, and rinsed several times in distilled water, alcohol-desiccated and mounted on glass slides using Naphrax® (Brunel Microscopes Ltd, Wiltshire, U.K.). Slides were examined with a Zeiss Axio Scope 100 A1 (Carl Zeiss, Jena, Germany) and Zeiss Axio Imager 2 (Carl Zeiss, Jena, Germany) light microscopes (LM), with differential interference contrast (DIC) optics at University of Perpignan and University of Szczecin, respectively. For the examination of raw or cleaned material by scanning electron microscopy (SEM), the samples were filtered through 1 µm Nuclepore® filters and rinsed twice with deionized (milliQ) water to remove salts. Filters were air-dried and mounted onto aluminum stubs before coating with gold-palladium.

### Electron microscopy (EM)

Ultrastructural analysis was made with scanning and transmission electron microscopy. For the SEM examination, a drop of the cleaned sample was filtered onto Whatman Nuclepore polycarbonate membranes (Fisher Scientific, Schwerte, Germany). The filters were air-dried overnight, mounted onto aluminum stubs, and coated with gold-palladium alloy (EMSCOP SC 500 sputter coater) and examined with a Hitachi S-4500 (Hitachi, Tokyo, Japan) field emission SEM operated at 5 kV, calibrated with a TGX01silicon grating (C2M) at University of Perpignan. Additional SEM observations were made at the Goethe University in Frankfurt am Main using a Hitachi S-4500 (Hitachi, Tokyo, Japan). For TEM observations, a drop of cleaned material was left to evaporate on a copper grid at room temperature. TEM observations were done at the Warsaw University of Technology, Faculty of Materials Science and Engineering, using a Hitachi SEM/STEM S-5500 (Hitachi, Tokyo, Japan), in which the specimens were simultaneously observed in scanning and transmission mode.

### Focus Ion Beam (FIB)

The FIB sectioning of the sternum valve was performed by means of a FIB/SEM Hitachi NB5000 integrated system. This system consists of an ultra-high performance focused Ga+ ion beam gun (40 kV) and high-resolution field emission gun scanning electron microscope (30 kV). This dual beam system enabled high-throughput specimen preparation, high resolution imaging and analysis, as well as precision nano-milling. During the FIB milling, particular parameters of ion beam conditions were selected to minimize the damage to our samples. Prior to cutting, a Tungsten (W) layer was applied for protection. The valve cut by FIB was selected from a sample taken from Juan de Nova from which the holotype of *Xenobennettella coralliensis* sp. nov. originated.

### Terminology and abbreviations

The terminology used follows Anonymous (1975), Ross et al. (1979) and Round, Crawford & Mann (1990). As previously proposed by Riaux-Gobin & Romero (2013) abbreviations are as follows: for the valves with a raphe, designated the raphe valve, is RV, for valves without a raphe, designated the sternum valve is SV; for the sternum valve valvocopula is SVVC, for the RV valvocopula is RVVC.

## Results

*Xenobennettella* Witkowski & Riaux-Gobin gen. nov.

**Description:** Frustules narrowly rectangular in girdle view with rounded corners. Valves elliptic to linear-elliptic with obtusely rounded apices. SV generally convex with concave sternum and unilateral central area. Cavum present on internal surface of central area. Transapical striae relatively robust, easily resolvable by LM. Alveolae open internally along valve margin as round foramina. SVVC with short, rectangular fimbriae on each side of foraminae. RV slightly concave with a somewhat elevated margin and raphe system. Valve mantle delicately striated (with pyramidal groups of small areolae). Sternum very narrow, raphe filiform—straight with external central endings approximate and slightly expanded, distal raphe endings (as shown by EM) bent in same direction. Internally, raphe slit opens laterally along elevated sternum with proximal raphe endings bent in opposite directions. RV transapical striae resolvable by LM only along the marginal part of valve. In EM, striae composed of triseriate rows of areolae along valve margin, becoming composed of loosely spread areolae towards raphe. Each stria delineated by a rather robust transapical rib (virgae). Areolae in both SV and RV occluded with hymenes bearing radiate slits. Internally, RV valve margin strongly bent inward, supported by transapical ribs that mark the transapical striae externally. Girdle composed of a few plain copulae.

**Typus generis:** *Xenobennettella coralliensis* Witkowski & Riaux-Gobin sp. nov.

**Habitat:** SE coast of Juan de Nova, reef slope of a shipwreck ca. 20m deep, sampled on 28 April 2009.

**Etymology:** *Xenobennettella* (prefix from ancient Greek *ξɛ*’ *ν*o *ς*: stranger, unusual) refers to characteristics that would be unusual in *Bennettella*, *i.e.*, the cavum in the SV and the lack of a peculiar asymmetric axial area reaching the RV valve margin.

**Comment:** *Xenobennettella* is the first genus with a cavum inhabiting the subtidal environment. It is particularly interesting that, until now, it has only been observed on coral reefs. In the type habitat, Juan de Nova, the genus was abundant in coral samples from 20 m water depths. As outlined in the etymology, the new genus can be misidentified with *Bennettella*. The SV is alveolate and similarly shaped in both genera. However, they can be distinguished by the presence of a cavum in *Xenobennettella*, which is absent in *Bennettella*. Moreover, the RV differs by simple symmetric raphe system and raphe branches bent in the same direction in *Xenobennettella* and sigmoid raphe branches with spectacular fascia in *Bennettella*. In fact, the new genus also shows some similarities to *Epipellis*, however, in the latter genus two valves are alveolate, whereas in *Xenobennettella* only the SV is alveolate.

*Xenobennettella coralliensis* Witkowski & Riaux-Gobin sp. nov.

**Figure 2:**
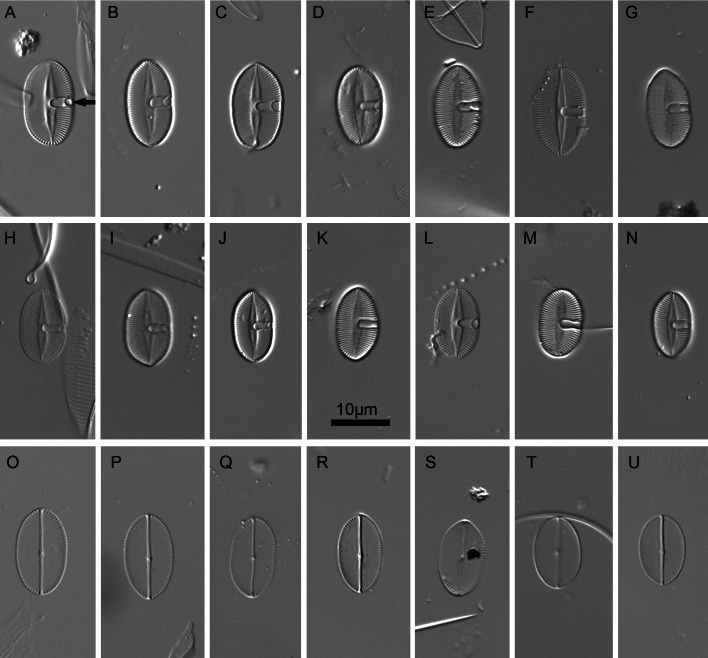
*Xenobennettella coralliensis* Witkowski & Riaux-Gobin sp. nov. imaged in light microscope. (A–N) The sternum valve—SV. (O–U) The raphe valve—RV. (A) Holotype specimen. (arrow in A) The presence of cavum.

**Holotype:** Fig. 2A: slide SZCZ16502 housed at the Institute of Marine and Environmental Sciences, University of Szczecin, Szczecin, Poland, leg. Dr. H. Breugemann University of La Reunion.

**Isotype:** Slide no. BM 81874 in Coll. of The Natural History Museum in London

**Type habitat:** sediment released in the ice box after removal of corals originally living 20m deep from the reef slope.

**Etymology:** the specific epithet refers to the occurrence of the new species on corals.

### Description

#### LM (Fig. 2)

Valves elliptic to linear elliptic, 11–15 µm long, 5.0–9.0 µm broad with broadly rounded apices. SV valves (Figs. 2A–2N), with distinct and lanceolate sternum, broad in the middle and becoming very narrow towards apices. Transapical striae distinct and resolvable by LM, parallel in the middle, becoming strongly radiate towards apices, 24–32 in 10 µm. In the middle part of valve unilaterally positioned distinct cavum. In RV valve (Figs. 2O–2U), raphe sternum very narrow but well resolved by LM. Raphe branches straight, central nodule distinct, proximal raphe endings dot-like, slightly expanded, distal raphe endings terminate at valve margin. Transapical striae resolvable only along margin, parallel in the middle becoming strongly radiate towards apices, 24–26 in 10 µm.

#### EM (Figs. 3–7)

**Figure 3:**
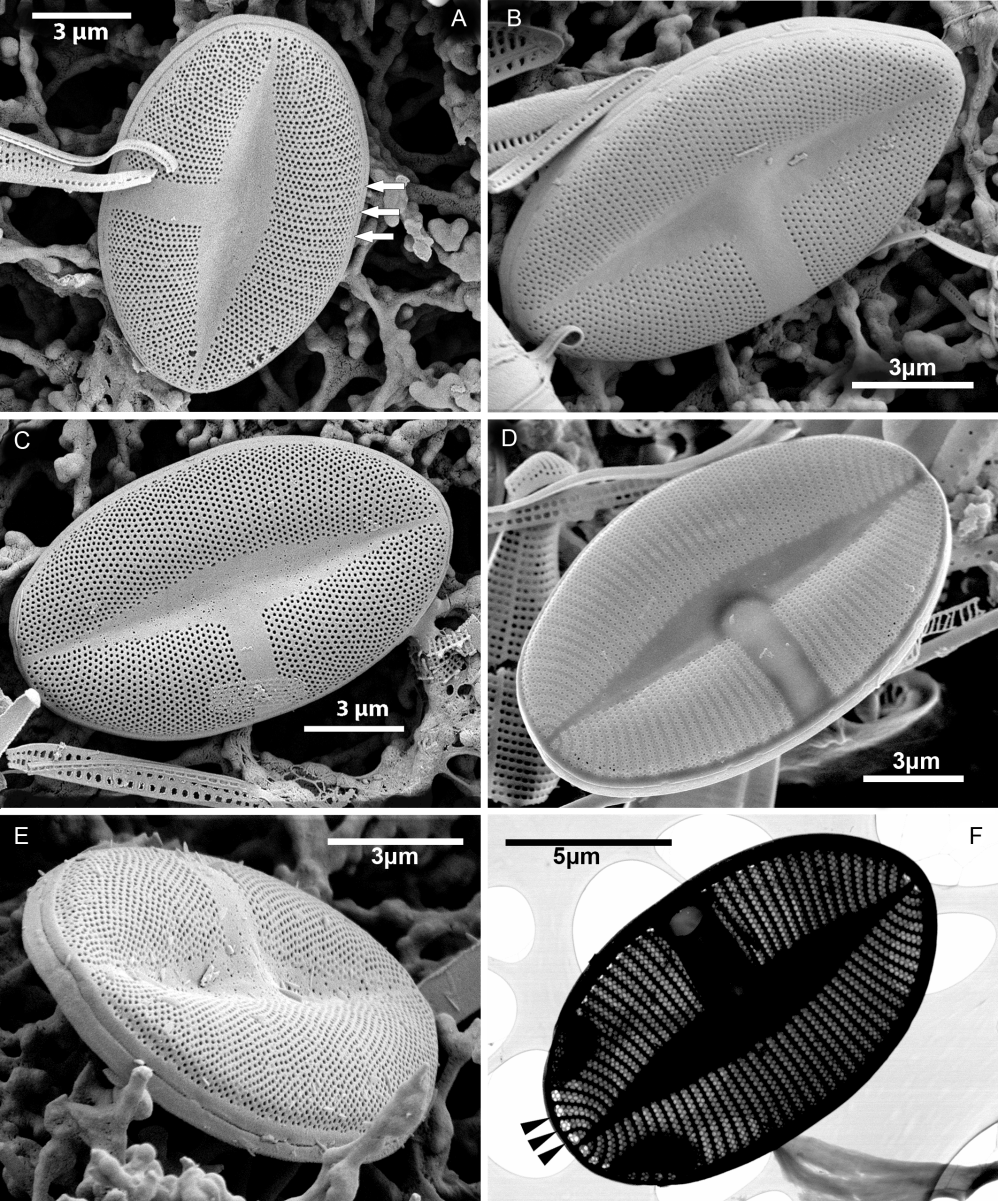
*Xenobennettella coralliensis* Witkowski & Riaux-Gobin sp. nov. SV external surface. (A–D) Lanceolate sternum and unilateral central area imaged in SEM. White arrows in A point the striae forming areolae positioned in quincunx between distinct virgae. (E) The external surface of broken SV with attached plain SVVC. (F) Openings of alveolae along the margin (black arrowheads).

**Figure 4:**
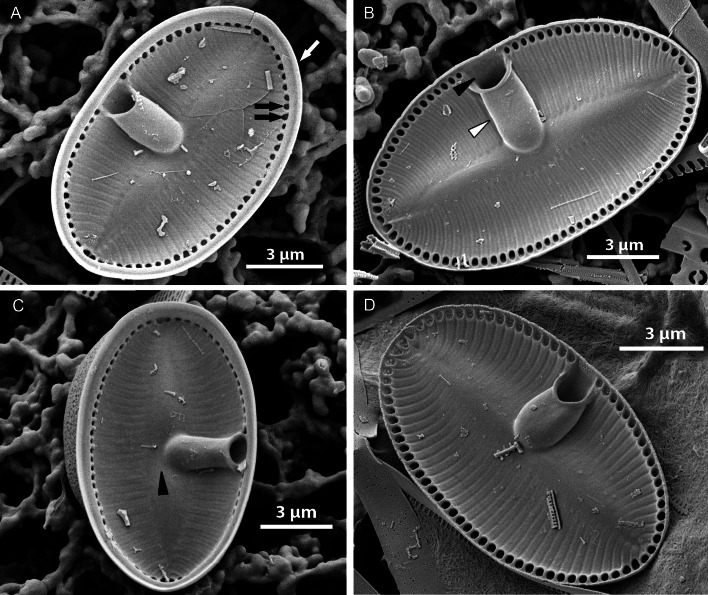
*Xenobennettella coralliensis* Witkowski & Riaux-Gobin sp. nov. SV internal surface imaged in SEM. (A) Internal valve surface with SVVC (white arrow) and alveolar openings (black arrows). (B) Valve interior devoid of SVVC with the position of cavum (white arrowhead) and the break in alveolae along the cavum entrance (black arrowhead). (C–D) Slightly elevated and plain sternum with a small depression around the contact with cavum (arrowhead in C).

Sternum valve (SV; Figs. 3, 4 and 7): valve external surface generally domed with narrow, plain mantle (Fig. 3B). Abrupt transition from valve surface to mantle. Sternum relatively broad, lanceolate in shape and concave, expanding towards valve centre into relatively broad unilateral central area (Figs. 3A–3F). Alveolate transapical striae parallel in the middle, becoming radiate towards apices, 24–32 in 10 µm with coarse transapical ribs (virgae) between stria (Fig. 3F). The transapical ribs connected with short and robust apically oriented viminae (Fig. 7C). Each alveola opens internally on margin by/via a round foramina (Figs. 7C and 7E). Externally, striae composed of biseriate rows of small, densely packed areolae (55–65 in 10 µm) that became triseriate rows towards valve margin, forming a quincunx pattern over whole SV surface. Areolae occluded by hymenes bearing radiate slits; hymenes positioned somewhat below valve surface (Figs. 6C–6D). Internally, alveolae covered with a structureless siliceous membrane and open into valve interior near valve mantle (Figs. 4 and 7C). The alveolae openings (or foraminae) small and oblong to elliptic in shape (Figs. 4 and 6A–6B). In the mid-valve interior, sealed to unilateral central area, distinctive relatively large cavum with a thin solid siliceous wall. Cavum interior flat and embedded with the same thin siliceous membrane that encloses the alveola in whole SV interior (Figs. 4, 6A, 7A and 7D). Girdle narrow composed of plain bands with narrow SVVC with short triangular fimbriae (Figs. 3D, 3E and 6B).

**Figure 5:**
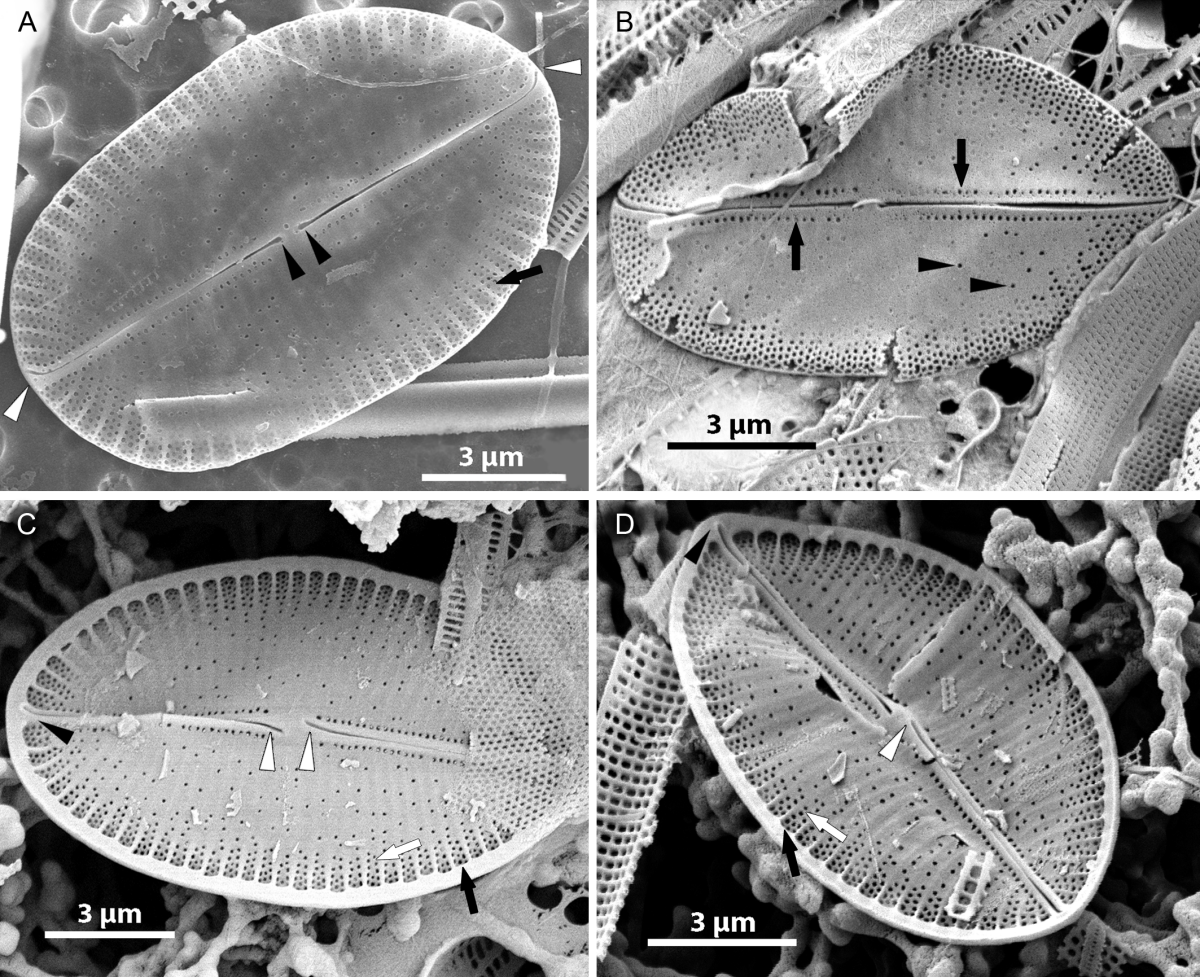
*Xenobennettella coralliensis* Witkowski & Riaux-Gobin sp. nov. RV imaged in SEM. (A–B) External and (C–D) internal surface of the RV. (black arrow in A) Transapical striae composed of bi- to triseriate areolae positioned along the valve margin. (black arrowheads in A) Raphe branches with slightly expanded proximal ends. (white arrowheads in A) Apical ends gently bent towards the valve margin. (black arrowheads in B) Valve middle with transapical striae marked only by solitary rows of areolae. (black arrows in B) Single row of areolae framing the raphe system. (black arrow in C–D) Internal view illustrating the chambered valve margin (black arrow on Figs. 5C–5D), (white arrow in C–D) Transapical striae with gradually decreasing areolae. (white arrowheads in C–D) Raphe branches with proximal raphe endings bent in opposite directions. (black arrowhead in C–D) Raphe terminating at apices in a small and indistinct helictoglossae.

**Figure 6:**
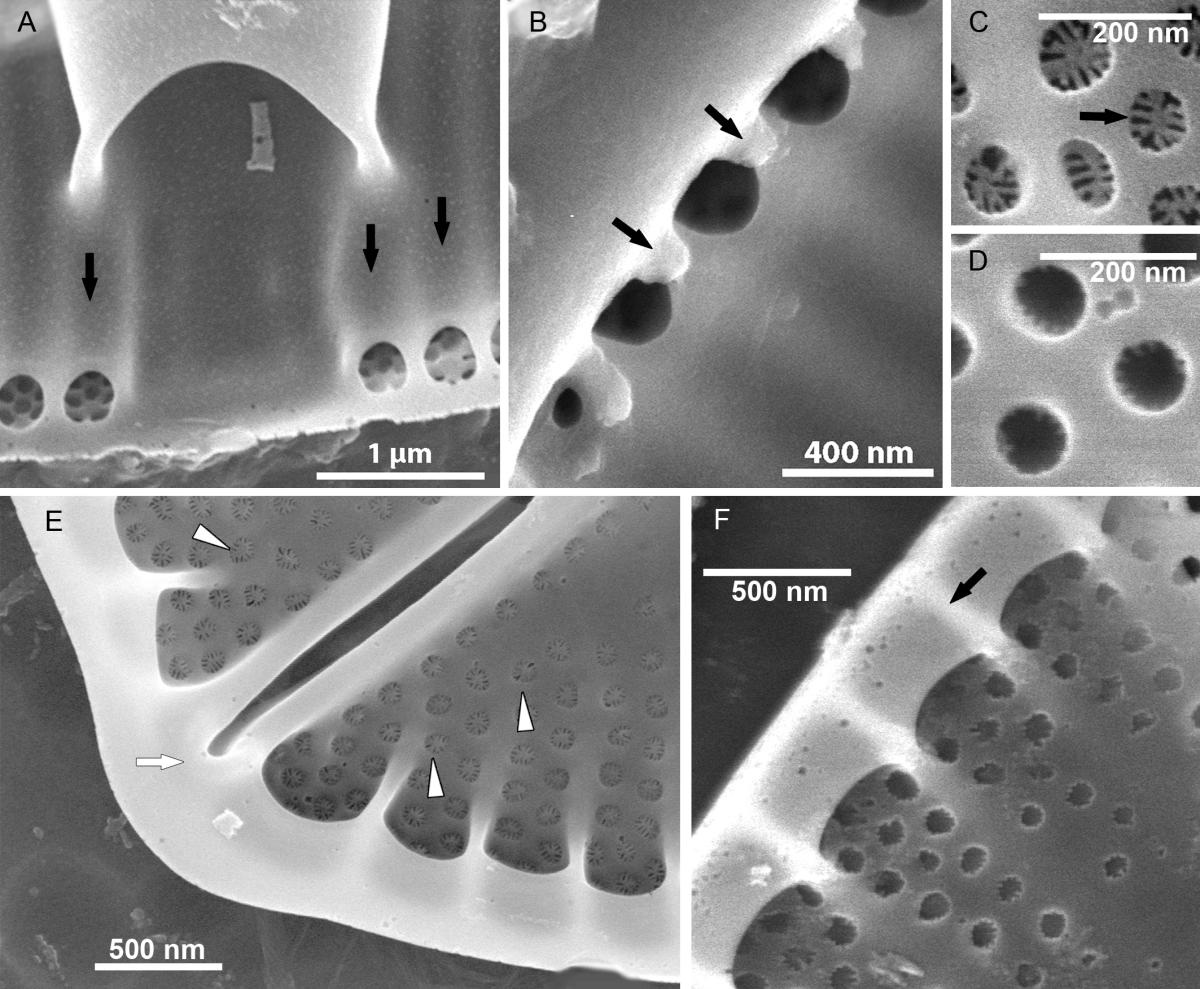
Details of *Xenobennettella coralliensis* Witkowski & Riaux-Gobin sp. nov. valve ultrastructure. (A–D) The internal view of the SV. (E–F) The internal view of the RV. (A) Close up of a break in alveolae along the cavum entrance (arrows point to alveolae). (B) Close up of the SVVC. (arrows in B) Triangular fimbriae between each alveolar opening on the. (C–D) SV external surface with (arrow in C) areola occlusions. (E) Interior of the RV with structures resembling chambers. (white arrow in E) Small and indistinct helictoglossa. (white arrowheads in E) areolae occlusions with slits. (F) Internal valve margin bent inwards. (arrow in F) Robust ribs between stria.

Raphe valve (RV; Fig. 5): valve external surface flat with somewhat elevated margins and raphe system. Sternum very narrow, separated from valve face by a row of small, densely packed areolae, up to 60–65 in 10 µm (Figs. 5B and 5D). Raphe filiform with external proximal raphe endings slightly expanded and approximate to each other (Fig. 5A). Apical raphe endings terminate on valve mantle (in a few cases observed below valve apex) and bent into the same direction (Figs. 5A–5B). Transapical striae close to valve margin composed of pyramidal groups of small areolae, grouped in triseriate rows and decrease towards valve center to biseriate or dispersed, solitary areolae (Figs. 5A–5B). On valve surface, each stria delineated by a slightly elevated rib that continues up to raphe sternum. Transapical striae at valve center parallel, becoming radiate towards apices, 24–26 in 10 µm. Areolae small, circular, internally occluded by hymenes bearing radiate slits, the same as observed in SV (Fig. 5E). Valve margin internally with structures resembling chambers, but open to cell lumen (Figs. 6E–6F). Moreover, internal valve margin strongly bent towards valve interior, with rather robust ribs between stria (Fig. 6A). Unfortunately, despite the numerous analyzed valves, we did not clearly observe the RV valvocopula (RVVC). The raphe system distinctly elevated, raphe slit opens laterally. Internal proximal raphe endings bent in opposite directions, whereas distal raphe ends terminate in small, simple helictoglossa at apices (Figs. 5C–5D).

**Figure 7:**
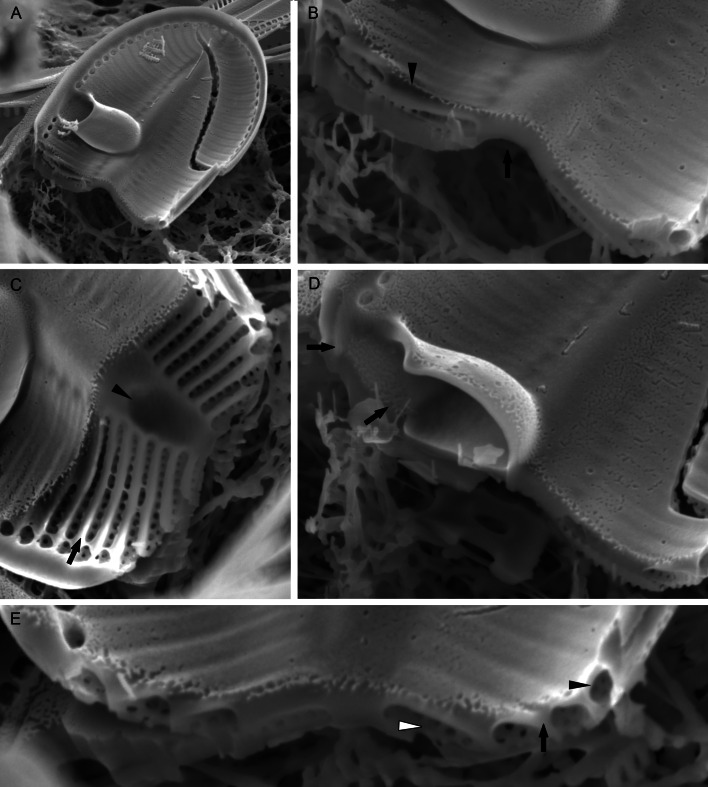
*Xenobennettella coralliensis* Witkowski & Riaux-Gobin sp. nov. Details of SV valve ultrastructure revealed with FIB nanocuts. (A) General view of SV cut with FIB. (B) Close up of the cut illustrated in A. (arrowhead in B) Internal valve surface membrane. (arrow in B) Solid sternum. (C) Removed with FIB valve interior membrane of the same specimen. (arrow in C) Robust virgae. (arrowhead in C) Depressed sternum. (D) Close up of the cut through the cavum. (arrows in D) Lack of alveolar openings within cavum solid structure. (E) Close up of the cut through alveolae. (arrow in E) Robust virgae. (Black arrowhead in E) Alveolar openings. (White arrowhead in E) Biseriate areolae of the striae.

## Discussion

### Taxonomic position of *Xenobennettella*

Since the publication of Round, Crawford & Mann (1990), monoraphid genera primarily included in two major and heterogenous *Achnanthes* sensu lato and *Cocconeis* sensu lato genera have been split into a number of genera based on the re-evaluation of distinctive and shared morphological characteristics. The major common feature of the latter taxa was their heterovalvy, with both a RV and a SV, while other characteristics *e.g.*, the girdle including the valvocopulae, orientation and shape of areola, valve outline or central area structure—showed a great deal of morphological variation. Taxonomic revisions of *Achnanthes* Bory and *Cocconeis* date back to the end of 19th century (*e.g.*, Cleve & Grunow, 1880), but continued into the 20th century (Holmes, 1985; Hustedt, 1933; Hustedt in Schmidt, 1937; Lange-Bertalot & Ruppel, 1980). Round, Crawford & Mann (1990) resurrected the “forgotten” genera, *e.g.*, *Achnanthidium* Kützing and *Eucocconeis* Cleve. However, the former genus was defined too broadly and further taxonomic work was required to conform the taxa included in this genus to the generitype *Achnanthidium microcephalum* Kützing. In the following papers, the diagnosis of *Achnanthidium* was refined further and several new genera established, including *Planothidium* Round & Bukhtiyarova, *Psammothidium* Bukhtiyarova & Round, *Karayevia* Round & Bukhtiyarova ex Round and *Kolbesia* Round & Bukhtiyarova ex Round (Round & Bukhtiyarova, 1996; Round, 1998). The process of transferring taxa from *Achnanthes* and *Cocconeis* sensu lato into morphologically appropriate, newly-established genera has continued over the last three decades and additional new genera have been established: *Lemnicola* Round & Basson, *Pogoneis* Round & Basson (Round & Basson, 1997), *Astartiella* Witkowski, Lange-Bertalot & Metzeltin, *Amphicocconeis* (De Stefano & Marino, 2003), *Scalariella* (Riaux-Gobin, Witkowski & Ruppel, 2012), *Gliwiczia* (Kulikovskiy, Lange-Bertalot & Witkowski, 2013) and *Madinithidium* Witkowski & Desrosiers (Desrosiers et al., 2014). Despite these revisions, small groups of achnanthoid and cocconeid taxa remain without detailed generic accommodation. An example of such a group is described here as a new genus *Xenobennettella*.

Our original sampling in the Scattered Islands involved only a collection of material preserved in 70% alcohol, which excludes any chance of the isolation of single specimens and establishing a clonal culture and thus the use of molecular markers. Its systematic position is based on several characteristics crucial for the monoraphid diatoms. Cox (2015) included Achnanthaceae in the Order Mastogloiales, whereas the remaining monoraphid clades were placed into the Order of Cocconeidales and two Families Cocconeidaceae and Achnanthidiaceae (cf. Round, Crawford & Mann, 1990). Taking into consideration the characteristics shared by *Xenobennettella* with other monoraphid genera, *i.e.*, (1) presence of sternum and cavum on one SV valve, (2) the chambered internal RV margin and alveolate SV and (3) internal proximal raphe ends bent in opposite directions on RV, plus areola occlusions with short radiate slits, we place the new genus in a clade with *Planothidium*, which belongs to Achnanthidiaceae (Cox, 2015).

### *Xenobennettella* key characteristics for identification

**Presence of cavum (1).** *Xenobennettella* is the third monoraphid genus bearing this structure and is the first purely marine organism with a cavum. In *Planothidium*, a cavum or rimmed depression in the SV valve occurs facultatively and has never been observed in marine forms (Riaux-Gobin et al., 2011; Riaux-Gobin, Compère & Al-Handal, 2011; Witkowski, Lange-Bertalot & Metzeltin, 2000). *Gliwiczia* includes purely freshwater forms but differs from the two other cavum bearing genera possessing it on both SV and RV (Kulikovskiy, Lange-Bertalot & Witkowski, 2013; Potapova, 2016). In all of the above listed genera, the presence of cavum is manifested on valve surface as a fascia. Among the particular genera, cavum shows 3-D structural differences. In *Xenobennettella*, the cavum expands from valve margin towards sternum and occupies valve inner surface corresponding to an external unilateral fascia. In *Planothidium*, cavum is more or less similar to *Xenobennettella* as shown in numerous examples illustrated by Jahn et al. (2017) and Stancheva et al. (2020). In *Planothidium* taxa, when present, it expands from valve margin towards sternum and its presence is marked by unilateral fascia on valve exterior. However, cavum is significantly different in *Gliwiczia* for which here we refer to as *Achnanthes calcar* Cleve—the name used by Montgomery (1978) when he observed the first ever SV of *Xenobennettella* in Florida waters. *Achnanthes calcar* has been transferred in *Gliwiczia* (Cleve) by Kulikovsky, Lange-Bertalot & Witkowski. As raised in Kulikovskiy, Lange-Bertalot & Witkowski (2013), but cf. Potapova (2016), no RV of *Achnanthes calcar* was illustrated in Cleve (1891). Fortunately, the original material studied by Cleve (1891) from Finland has been sent in the past to the National Academy of Sciences in Philadelphia and in slides made from this material RV of *Achnanthes* (*Gliwiczia*) *calcar* was illustrated (cf. Kulikovskiy, Lange-Bertalot & Witkowski, 2013; Potapova, 2016). The cavum in *Gliwiczia* and in *G. calcar*, in particular, is different from *Xenobennettella* and *Planothidium*, firstly occurring on both RV and SV, secondly it is rather small and made of a thick silica layer. It extends from the valve margin to ca. $ \frac{1}{2} $ distance to mid sternum. An internally terminating cavum prolongs into a distinct and elevated fascia that shows a decrease towards the opposite valve margin. Like in *Xenobennettella* and *Planothidium*, cavum in *Gliwiczia* is expressed externally, but in the latter genus on valve face as a fully developed fascia with a somewhat diminishing size towards the opposite valve margin. This is completely different when compared to *Planothidium* and *Xenobennettella* that both have unilateral fascia. With the unique major characteristics presented above for *Xenobennettella*, as typified by *X. coralliensis*, this is an interesting discovery. This new diatom genus was found in the sublittoral zone of coral reefs of the Indo-Pacific, habitats that are fertile grounds for undiscovered biodiversity and diatom morphologies (*e.g.*, Al-Handal, Compère & Riaux-Gobin, 2016; Kryk et al., 2021; Lobban et al., 2012; Riaux-Gobin, Compère & Al-Handal, 2011; Riaux-Gobin, Compère & Al-Handal, 2011; Risjani et al., 2021). The diatom genus we studied bears a set of characteristics previously seen in monoraphid genera from very different ecologies: the freshwater *Planothidium* and *Gliwiczia* and the epizoic *Bennettella* and *Epipellis* (Denys & De Smet, 2010; Ferrario et al., 2019). Many monoraphid diatom species are characterized by the presence of a horseshoe (based on a German term Hufeisen), but renamed recently as hood or cavum (https://diatoms.org/genera/planothidium/guide). SEM observations show that this structure is a rimmed depression resembling a cave. The first cavum structures were observed in *Achnanthes* taxa, which are now transferred to *Planothidium* Round & Bukhtiyarova and *Gliwiczia* Kulikovskiy, Lange-Bertalot & Witkowski. Whereas *Planothidium* possesses a cavum only on the SV, *Gliwiczia* species possess it on both valves (Krammer & Lange-Bertalot, 1988; Kulikovskiy, Lange-Bertalot & Witkowski, 2013; Round, Crawford & Mann, 1990). In terms of habitat, all known taxa bearing a cavum have been recorded in freshwater (most *Gliwiczia* occur in Lake Baikal) or slightly brackish-waters (*Planothidium*), making *Xenobennettella* the first exclusively marine taxon to bear this structure with a specific coral reef habitat from the Indo-Pacific and Florida Keys. The cavum structure has been observed thus far only in *Planothidium* (in some species) and *Gliwiczia* (in all species; Table 1).

**Table 1:** Morphological comparison of *Xenobennettella* gen. nov. with similar genera.

**Character**	***Xenobennettella***	***Bennettella***	***Epipellis***	***Gliwiczia***	***Planothidium***
Raphe valve	Monolayered	Monolayered	Alveolate	Monolayered	Monolayered
Raphe system	Straight	Slightly sigmoid	Sigmoid	Straight	Straight
Raphe external proximal ends	Simple, slightly expanded	Simple, slightly expanded	Simple, slightly expanded	Simple, slightly expanded	Simple, slightly expanded
Raphe external apical ends	Bent in the same side, terminate on the apical mantle	Terminate below apices under a siliceous triangle	Expanded, terminate below apices	Simple slightly bent in opposite directions, terminate on apieces	Bent in the same side, terminate on the apical mantle
Raphe internal proximal ends	Bent into opposite sides	Bent into opposite sides	Bent into opposite sides	Bent into opposite sides	Bent into opposite sides
Raphe internal apical ends (helictoglossae)	Small helictoglossae	No helictoglossae	No helictoglossae	Small helictoglossae	Small helictoglossae
RV central area (fascia)	Absent	Displaced two arms reaching the valve margin	Displaced two arms reaching the valve margin	Slightly asymmetric fascia	Variable in shape CA
RV cavum	Absent	Absent	Absent	Absent	Absent
RV mantle	Plain	Structured	Structured	Areolated	Plain
RV internal margin	Chambered	Chambered	With alveola openings	Plain	Plain
RV transapical striae	Bi- to triseriate on margin in the middle dispersed	Biseriate	Biseriate	Uniseriate	Uni- to triseriate
Areola occlusions	Hymenes with slits	ND	ND	ND	Hymenes with slits
Sternum valve structure	Alveolate	Alveolate	Alveolate	Monolayered	Monolayered
Sternum	Lanceolate to linear-lanceolate	Very narrow, sigmoid	Narrow linear	Linear	Linear to linear- lanceolate
SV central area	Unilateral	Absent	Absent	Bilateral	Occasionally unilateral
SV cavum	Always present	Always absent	Always absent	Always present	Occasionally present
SV transapical striae	Tri- to biseriate	Biseriate	Biseriate	Uniseriate	Uni- to triseriate
Areola occlusions	Hymenes with slits	ND	ND	Membranes with unresolved ultrastructure	Hymenes with slits

**Valve structure (2).** Whereas in LM the new genus due to the presence of a cavum may resemble *Planothidium* (but also with the proximal internal raphe endings bent in opposite directions and hymenate areolae occluded with slits), its SV alveolate structure eliminates the taxa from the latter genus. *Xenobennettella coralliensis*, as the first representative of the genus has clearly chambered internal RV margin and alveolate SV (Table 1), which rules out any resemblance between the two genera. Our newly described genus shares several features with *Bennettella* with regards to the alveolate SV ultrastructure. In both genera, the alveolae are internally closed with solid siliceous coverings and possess marginal foraminae. Comparing to *Bennettella* the key is also transapical striae of the SV and RV of *Xenobennettella*, which are composed of small areolae arranged in bi- to triseriate rows with each stria delineated by a transapical rib visible on the valve surface and interior. It is worth to remember, that internally, the raphe sternum is elevated over the valve surface in both genera, with the raphe slit opening laterally (see Ferrario et al., 2019). In addition, in the *Bennettella* RV, the fascia is complete over the whole valve (bilateral) and axially distorted, which is simple in *Xenobennettella*. Interestingly, *Xenobennettella* is also the first record of this structure associated with an alveolate valve, as *Gliwiczia* and *Planothidium* are both characterized by a simple porous valve ultrastructure.

**Raphe system (3).** The last major difference between other genera and our newly described genus is the RV raphe system. In other similar genera, the raphe is sinusoid and terminates below the apices whereas in *Xenobennettella* it is straight and its apical ends are bent to the same side at the apices with raphe branches terminating on the valve mantle (externally; Table 1).

### Distribution and ecology

*Xenobennettella* was common in coral sand from 20 m deep at Juan the Nova, a coral island in the Mozambique Chanel, west of Madagascar, but also observed in Florida (Montgomery, 1978, Pl. 3: D). Montgomery (1978) imaged a single SV of *Xenobennettella coralliensis* from the coral sand of Florida Keys and identified it as *Achnanthes calcar*. Due to the fact that *Achnanthes* (*Gliwiczia*) *calcar* inhabits freshwaters, Montgomery possibly considered this taxon as redeposited from a terrestrial habitat. Thus far we have observed two species of *Xenobennettella* with *X. coralliensis* in Juan the Nova Island of the Western Indian Ocean and possibly a second species in the Tuamotu Archipelago in the South Pacific.

Our research continues to document the seemingly independent evolution of many ultrastructural characteristics across diatoms. Apparently, the cavum in *Xenobennettella* is possibly another example of homoplasy in diatom valve ultrastructure, along with the alveolate cross-section. With the documented paraphyly of monoraphid diatoms (Davidovich et al., 2017; Górecka et al., 2021; Riaux-Gobin et al., 2021), we are hopeful that we will be able to test in the future the hypothesis that this genus may represent a secondary gain of the monoraphid frustule close to the pinnularioid diatoms, based on similarities in the alveolate valve ultrastructure.

## Conclusions

In this article we formally describe the new monoraphid genus characterized with monolayered raphe valve with raphe branches terminating on the valve mantle (externally), proximal raphe ends bent in opposite directions internally and the alveolate sternum valve. *Xenobennettella* is the first described marine diatom genus characterized with a cavum. Likewise, this is the first cavum-bearing diatom genus with alveolate sternum valve. The newly described genus *Xenobennettella* was observed in the coral reef habitats in the Mozambique Channel in the Indian Ocean and Tuamotu Archipelago in the SE Pacific, but in the past, it has been illustrated from the Florida Keys in the tropical Atlantic Ocean.

## Additional Information and Declarations

### Data Availability

The following information was supplied regarding data availability: The article describes a new diatom genus and species and therefore there is no dataset for this taxonomical work. All data are presented in the article.

### New Species Registration

The following information was supplied regarding the registration of a newly described species: New diatom genus: Xenobennettella Witkowski & Riaux-Gobin gen. nov. and new diatom species: *Xenobennettella coralliensis* Witkowski & Riaux-Gobin sp. nov.
